# Lipid rafts and human diseases: why we need to target gangliosides

**DOI:** 10.1002/2211-5463.13612

**Published:** 2023-04-20

**Authors:** Jacques Fantini

**Affiliations:** ^1^ Aix Marseille Univ, INSERM UMR_S 1072 Marseille France

**Keywords:** Alzheimer, cancer, ganglioside, lipid raft, Parkinson, virus

## Abstract

Gangliosides are functional components of membrane lipid rafts that control critical functions in cell communication. Many pathologies involve raft gangliosides, which therefore represent an approach of choice for developing innovative therapeutic strategies. Beginning with a discussion of what a disease is (and is not), this review lists the major human pathologies that involve gangliosides, which includes cancer, diabetes, and infectious and neurodegenerative diseases. In most cases, the problem is due to a protein whose binding to gangliosides either creates a pathological condition or impairs a physiological function. Then, I draw up an inventory of the different molecular mechanisms of protein‐ganglioside interactions. I propose to classify the ganglioside‐binding domains of proteins into four categories, which I name GBD‐1, GBD‐2, GBD‐3, and GBD‐4. This structural and functional classification could help to rationalize the design of innovative molecules capable of disrupting the binding of selected proteins to gangliosides without generating undesirable effects. The biochemical specificities of gangliosides expressed in the human brain must also be taken into account to improve the reliability of animal models (or any animal‐free alternative) of Alzheimer's and Parkinson's diseases.

## Graphical abstract

Membrane gangliosides are involved in many diseases, often through a modification of lipid raft homeostasis due to the binding of a pathological protein. Thus, these diseases should be considered as membrane disorders. This article develops the ‘ganglioside‐targeting therapy’ concept for treating such diseases with innovative molecules capable of selectively disrupting the binding of pathological proteins to gangliosides.

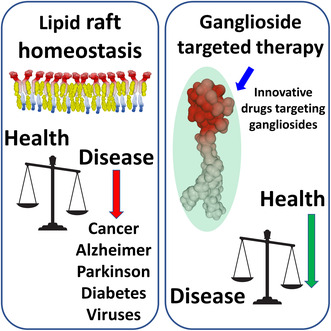

**Abbreviations**
BBB: blood–brain barrier
Covid‐19: coronavirus disease 2019
GBD: ganglioside‐binding domain
Neu5Ac: *N*‐acetylneuraminic acid
Neu5Gc: *N*‐glycolylneuraminic acid
NTD: N‐terminal domain
RT‐PCR: reverse transcription polymerase chain reaction
SARS‐CoV‐2: severe acute respiratory syndrome coronavirus 2
SNARE: soluble *N*‐ethylmaleimide‐sensitive factor attachment protein receptor

As surprising as it may seem, the definition of the term ‘disease’ is not obvious [1]. The current Covid‐19 pandemic is a blatant example of this. This infectious disease, fatal for some, can be mild for others and sometimes completely unnoticed. But the RT‐PCR screening tests for SARS‐CoV‐2 have been able to maintain the confusion between people positive for the virus, who may not show any symptoms, and patients with symptomatic Covid‐19. The same applies to hypercholesterolemia or hypertension, which are more risk factors for disease and not symptoms by themselves. What is and what is not a disease has been the subject of interesting discussions [2], and the answers of people to whom the question was asked could have been very surprising. In general, infections such as tuberculosis or malaria are considered illnesses, while malnutrition, barbiturate overdose, carbon monoxide poisoning, or skeletal fractures are considered illnesses only by 20% of people [3]. We can welcome (or not) the fact that aging was classified as number 1 in the top 20 nondiseases by readers of the *British Medical Journal* [3]. However, the debate is not closed, and we can even say that the fight is fierce between those who regard aging as a disease and those who refute this association [4, 5, 6, 7, 8]. ‘Le temps ne fait rien à l'affaire’ (dixit the french poet and singer Georges Brassens), but now as years went by, I would tend to join the camp of those who do not consider aging as a disease. Anyway, this debate is relevant to the subject of this review because among the age‐related biochemical modifications, the expression of gangliosides holds a preponderant place [9, 10], in particular at the brain level [11, 12]. This is no way pathological by itself, but a condition that could facilitate a loss of function [13, 14, 15, 16] or a gain of toxicity [17, 18, 19], in association with other factors such as amyloid proteins [20]. I see this situation as a police investigation leading to the identification of culprits (the proteins) and accomplices (the gangliosides of the rafts). By neutralizing the accomplices, we can hope to prevent the crime, i.e., the disease. This explains why gangliosides [21] and/or drugs targeting gangliosides [22] should be considered as possible therapeutic compounds for a broad range of human disorders, including neurodegenerative diseases.

## Membrane diseases and gangliosides

The fundamental unit of the living world is the cell. Any disruption of cell function is therefore likely to induce a pathological condition [23]. The plasma membrane, which is positioned at the interface between the cell and its environment, is a privileged place for communication and the transfer of biological information. It is also at its level that pathogens make their way to infect a living organism. Overall, the components of the plasma membrane have the function of establishing a selective barrier between the cell and its environment, and also of ensuring communication with it. Gangliosides, as key operators of signal transduction pathways [24, 25, 26, 27], are at the forefront of pathological dysfunctions [28, 29, 30, 31]. In this respect, they may represent a privileged target for a new class of drugs [32, 33].

The principal diseases that can be classified as membrane disorders are listed in Table 1. As can be seen in this summary, gangliosides are systematically involved in all these diseases. In most cases, the problem is due to a protein whose binding to gangliosides either creates a pathological condition [34] or alters a physiological function [35]. Understanding the role of gangliosides in each pathology is a prerequisite to design specific drugs based on ganglioside structures for innovative therapeutic approaches. To this end, we must first analyze how gangliosides physiologically behave in the plasma membrane, and how the pathological conditions affect this behavior.

**Table 1:** Principal membrane disorders involving gangliosides.

Disease	Plasma membrane disorder (references)	Involvement of gangliosides (references)
Alzheimer's disease	[36, 37, 38, 39]	[31, 40, 41]
Parkinson's disease	[42, 43]	[29, 44, 45]
Creutzfeldt‐Jakob/Prion disease	[46, 47]	[48, 49]
Rett syndrome	[50, 51]	[52, 53]
Cancer	[54, 55]	[30, 56, 57]
Type 2 diabetes	[58, 59]	[60, 61]
Cystic fibrosis	[62, 63]	[64, 65]
Virus (and virotoxin) diseases	[66, 67, 68, 69]	[70, 71, 72, 73]
Bacterial (and bacterial toxins) diseases	[74, 75, 76, 77]	[78, 79, 80, 81]
Parasite diseases	[82, 83]	[84, 85]

## Ganglioside biochemistry and membrane biology

**Fig. 1:**
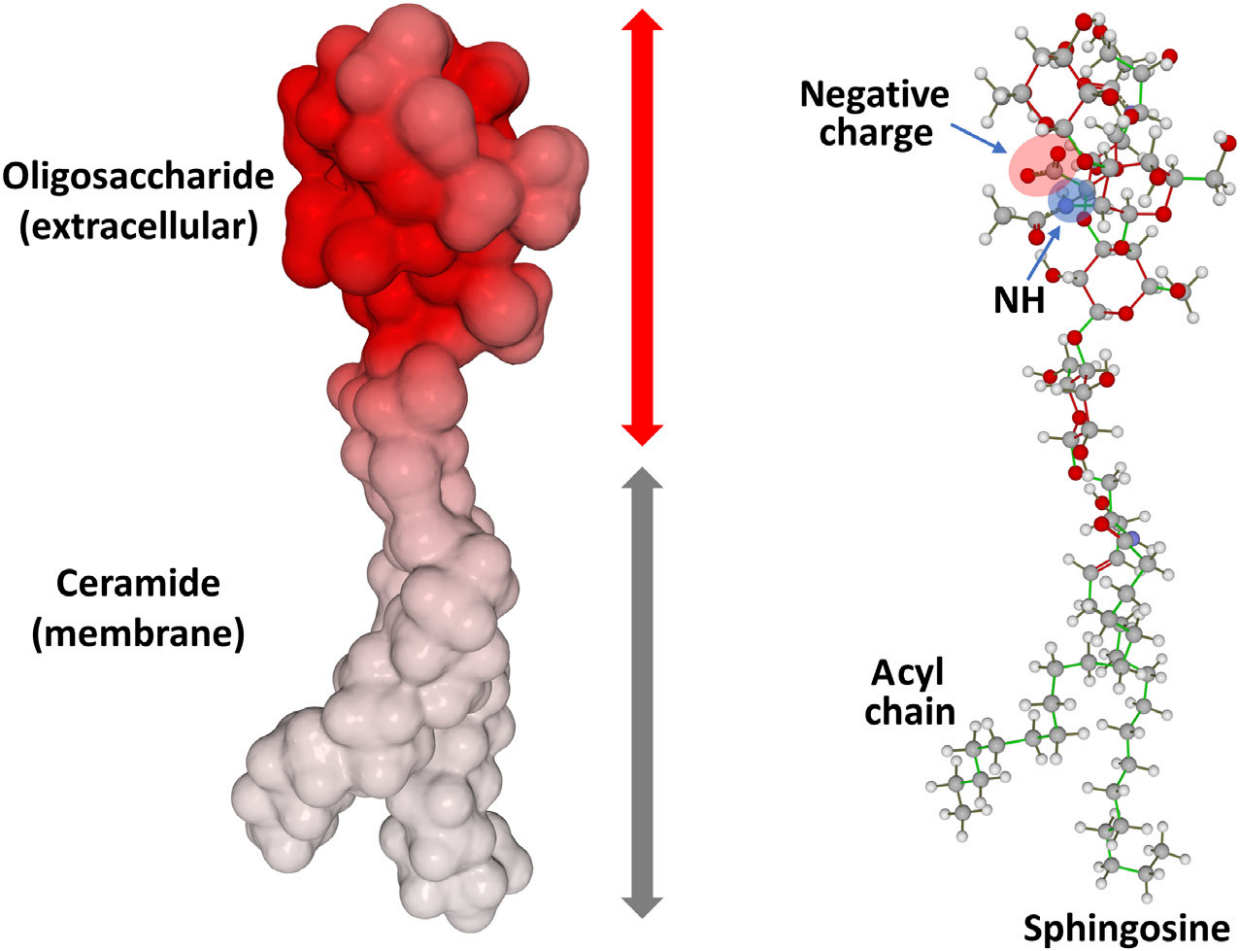
Structural properties of gangliosides. Left panel: electrostatic surface potential of ganglioside GM1 (red, electronegative; white/gray, apolar). Right panel: structural organization of ganglioside GM1. In the extracellular oligosaccharide part, the negative charge of sialic acid is neutralized by the NH of the amide group of *N*‐acetylgalactosamine. Ceramide (sphingosine + acyl chain) is embedded in the apolar part of the plasma membrane.

Gangliosides are glycosphingolipids containing at least one sialic acid [86]. At pH 7, they therefore have a negative charge (Fig. 1). It may therefore seem somewhat paradoxical that these gangliosides are not subject to electrostatic repulsion and instead group together in condensed clusters referred to as lipid rafts [87]. However, these associations are stabilized by two mechanisms: (a) in the apolar part of the membrane, by a strong interaction with cholesterol and (b) at the extracellular level, by the amide group of N‐acetylated sugars, which neutralizes the negative charge of sialic acids (Fig. 1). This particular effect, coined ‘NH trick’ by Azzaz et al. [86] is operative for GM1, GD1a, GD1b, and GT1b gangliosides, which are the four main gangliosides expressed in adult human brain [88]. It does not apply for smaller gangliosides (GM3 and GM4) that are lacking N‐acetylated sugars but also display the less intense electrostatic field (in addition to the sialic acid, GM3 has two sugars and GM4 only one sugar, whereas GM1 has four sugars including *N*‐acetylgalactosamine). The other sphingolipids that segregate in lipid rafts are sphingomyelin (a zwitterionic lipid displaying a positive and a negative electric charge) and neutral glycosphingolipids such as GalCer [87]. Sphingomyelin molecules can interact electrostatically by charge complementarity and the sugar headgroups of glycosphingolipids by hydrogen bonds. Thus, each raft lipid has its own way to minimize repulsion forces and favor attractive forces. The consequence is that the conformational freedom of each raft lipid is the result of a balance between attraction and repulsion, a property that is highly dependent on the local environment and can thus vary as a function of time.

**Fig. 2:**
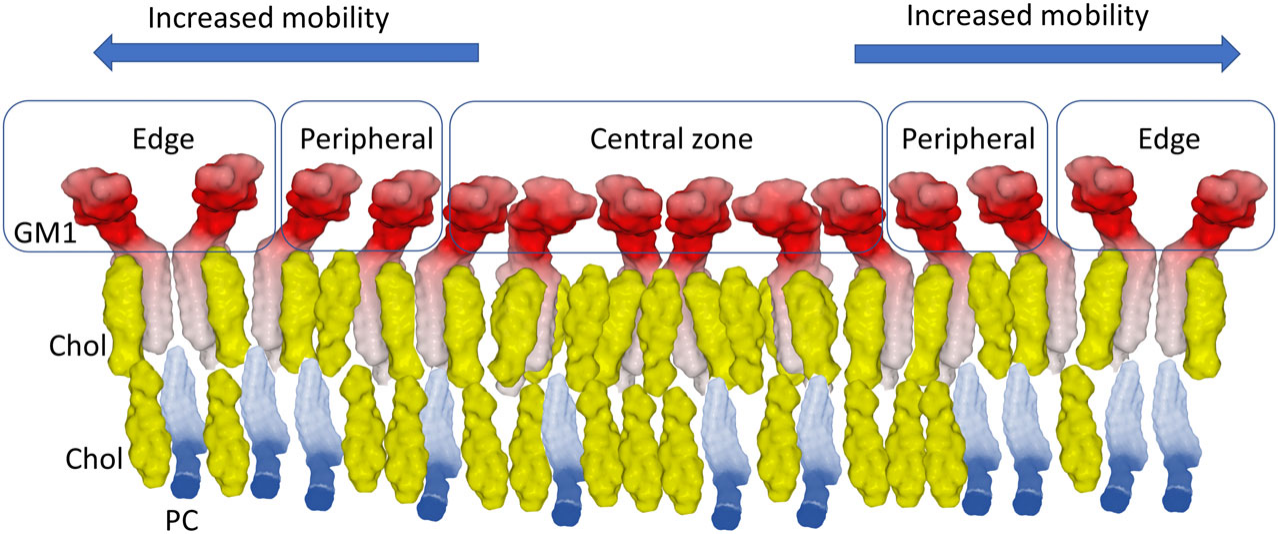
Different patterns of ganglioside distribution in lipid rafts. Not all gangliosides have the same level of mobility within rafts. This distribution is illustrated here with a raft containing ganglioside GM1. In the central zone, the gangliosides are very condensed and therefore have reduced mobility. When one approaches the edge of the raft, the gangliosides have higher degrees of freedom (peripheral zone). Finally, the most mobile gangliosides are localized on the edge of the rafts where they can form typical chalice‐like structures.

According to recent molecular dynamics simulation data, three different subpopulations of gangliosides (GM1) have been characterized in the same lipid raft [22]. These patterns of ganglioside distribution in lipid rafts are illustrated in Fig. 2. The gangliosides located in the central zone of the raft have reduced mobility because they are constrained by their neighbors. By contrast, peripheral gangliosides have more conformational freedom, and they can modify their shape when they interact with protein ligands. Finally, the gangliosides at the edge of the raft can adopt a typical chalice or butterfly‐like (open wings) dimeric conformation [89]. These conformational possibilities are further extended by the biochemical diversity of gangliosides [20]. In this respect, it is striking how gangliosides can seem so similar and at the same time so different. Their common points are the same backbone structure (sphingosine and fatty acid embedded in the apolar core of the plasma membrane) and the presence of at least one negative charge in their extracellular part. The concentration of these negative charges in the same raft creates a large negative electrostatic surface potential, which is one of the essential properties of lipid rafts [90, 91]. Thus, any protein, toxin, or pathogenic agent will bind all the more quickly and easily to a raft that it will have an electropositive potential [92]. However, the simplicity of this charge complementarity masks the subtlety of protein‐ganglioside interactions, which also take into account the conformational flexibility of gangliosides. This duality sums up the main difficulty of designing therapeutic molecules recognizing gangliosides and capable of competitively inhibiting the binding of pathogenic proteins to lipid rafts. In this respect, one important question to ask is against which ganglioside monomer, dimer, or cluster do we need to design an antiganglioside drug? To solve this problem, we must first study how proteins bind to gangliosides.

## Ganglioside‐binding domains: a proposed classification

Schematically, proteins that bind plasma membrane gangliosides can be divided into two categories: host plasma membrane proteins and extracellular proteins (either from the host or from a pathogen). This dichotomy generates de facto two molecular mechanisms of protein‐ganglioside interactions. But there is more, as we will see in this section. Let us first consider how the extracellular part of plasma membrane proteins interacts with gangliosides. Whether the protein has single or multiple transmembrane domains, the same rules should apply. First, this domain must be relatively flexible to be able to reach the ganglioside, and this is all the more so as the protein is constrained by its part immersed in the membrane. It should be remembered that gangliosides are generally associated with cholesterol molecules, which also help to stabilize the association of the protein with the plasma membrane [93]. Even if the role of cholesterol‐binding domains is outside the scope of this article, we cannot neglect the indirect role of raft‐associated cholesterol, which will influence the very nature of ganglioside‐binding domains [93, 94]. Flexibility opposing the rigidity of a well‐ordered three‐dimensional structure, the consequence is that a ganglioside‐binding domain is generally unfold (or at least partially unfold) prior to its interaction with a ganglioside. For this reason, gangliosides may exert a chaperone activity, constraining the protein to adopt a well‐defined structure following ganglioside binding [95, 96]. This is a simple and reversible way to regulate receptor function [95, 97]. For geometrical reasons, this may occur preferentially at the edge of a lipid raft, or even outside a lipid raft if a single ganglioside is extracted from the raft by the protein with which it will form a stabilized complex. A representative example is given by synaptotagmin, a synaptic vesicle protein that acts as a plasma membrane receptor for botulinum toxin B [77]. The extracellular domain of synaptotagmin is a region that is highly flexible and intrinsically disordered [98, 99]. Circular dichroism studies indicated that the ganglioside GT1b induced the α‐helical folding of synaptotagmin, which incidentally is the structure recognized by the botulinum toxin [77]. Hence, ganglioside GT1b is a mandatory host cofactor that renders synaptotagmin competent for botulinum toxin binding. The toxin also has a second site that can bind gangliosides independently of synaptotagmin, according to a dual receptor mechanism [100, 101].

**Fig. 3:**
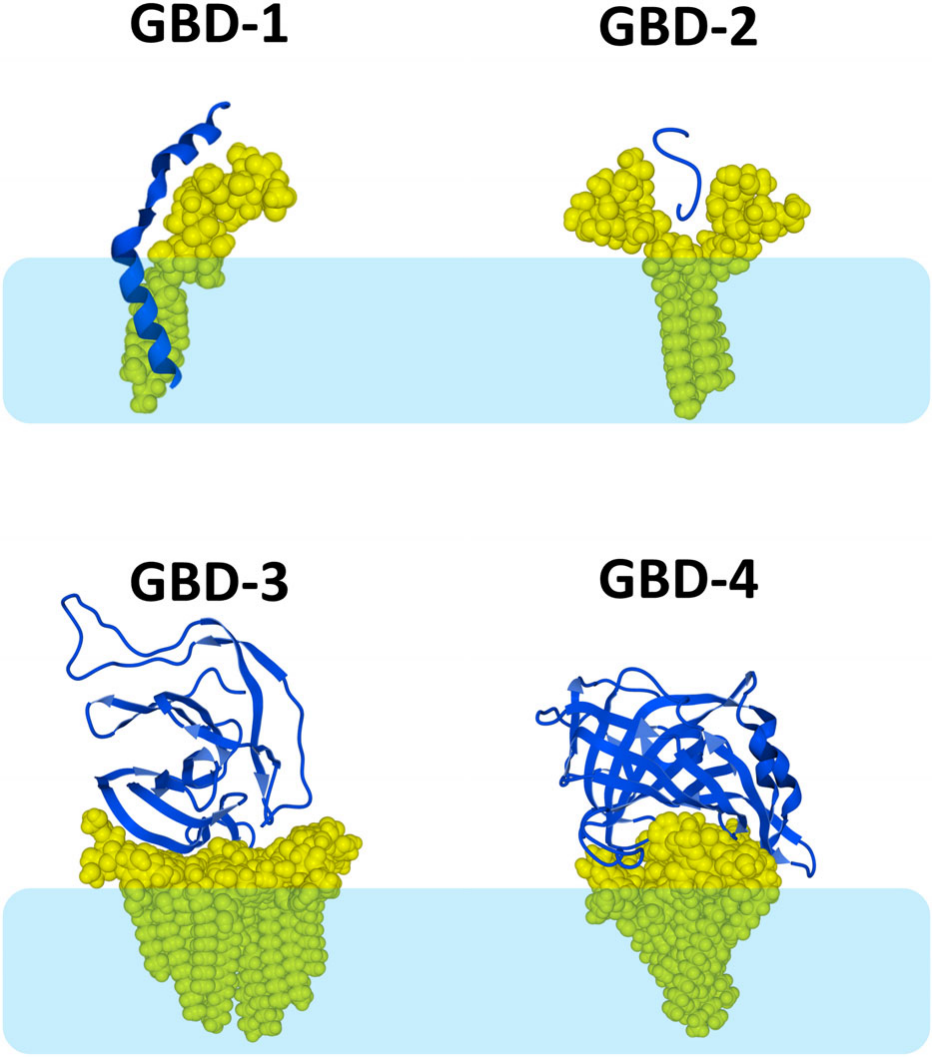
Classification of ganglioside‐binding domains. GBD‐1, synaptotagmin‐GT1b complex; GBD‐2, amyloid protein bound to a GM1 dimer; GBD‐3, SARS‐CoV‐2 N‐terminal domain (NTD) bound to a GM1 raft; GBD‐4, Monkeypox virus E8L protein bound a GM1 raft. The gangliosides are represented as yellow atomic spheres. Proteins are represented as blue ribbons, helices, turns, and coils. The light blue frame represents the apolar part of the plasma membrane (extracellular leaflet).

The chaperone effect of gangliosides on the extracellular domain of synaptotagmin probably has a physiological function linked to the biology of synaptic vesicles. Indeed, SNARE complex formation involves disordered proteins (synaptobrevin, syntaxin, and SNAP‐25) that undergo structuration upon binding to their partners [99, 102]. I propose to call type 1 GBD, or GBD‐1 (Fig. 3, upper left), any ganglioside‐binding domain able to form a stoechiometric (1 : 1, mol : mol) complex with a single ganglioside molecule. In the membrane receptology field, GBD‐1 generally belongs to a flexible juxtamembrane region that can switch from a disordered state to an ordered structure under the control of a ganglioside acting as a molecular chaperone. Because they interact with transmembrane glycoproteins, the gangliosides recognized by those GBD‐1 are expected to reside at the edge of a lipid raft, or in the immediate environment of a raft. These regions are in contact with the Ld phase of the membrane, which has an increased fluidity [87]. Other examples of membrane proteins displaying a GBD‐1 include the serotonin 5‐HT1A receptor [103, 104] and the tumor stem cell marker CD133 (prominin‐1) [105]. Further potential candidates are EGF and PDGF receptors, as well as ion transporters [88], which are also regulated by gangliosides [106]. The formal characterization of a potential GBD‐1 in these membrane proteins is in progress.

The second type of ganglioside‐binding domain is a dimeric structure resembling a flower chalice or the open wings of a butterfly [89, 107]. In this case, the protein must open the chalice to interact deeply with the ganglioside, starting a typical membrane insertion process [107]. The interaction is generally mediated by a hairpin loop of the protein (either pre‐existing or created during the binding reaction) that fits within the ganglioside chalice. The targeted gangliosides should have sufficient conformational flexibility to accommodate this loop, as it would be the case at the edge of a lipid raft but not in more central areas [22]. Thus, it is at the edge of a lipid raft that ganglioside‐dependent membrane insertion processes generally occur. The fusion of HIV‐1 with host cell membranes [68, 108] and the formation of oligomeric Ca^2+^ permeable amyloid pores [109, 110], both ganglioside‐dependent mechanisms, require such chalice‐shaped ganglioside dimers. Incidentally, gangliosides exert a chaperone effect on amyloid proteins that also belong to the category of intrinsically disordered proteins [110, 111, 112, 113]. I propose to name this second type of ganglioside‐binding domain type 2 or GBD‐2 (Fig. 3, upper right). GBD‐1 and GBD‐2 are short linear fragments of a protein and can thus be described by a consensus sequence motif [89].

A third type of ganglioside‐binding domain has emerged with SARS‐CoV‐2, the coronavirus responsible for the current Covid‐19 pandemic. It is a large and discontinuous flat surface region of the Spike glycoprotein that can lie on the lipid raft, ensuring the first contact and further reinforcement of the viral particle adhesion on the host cell membrane [68]. The binding of the Spike protein to a lipid raft involves numerous gangliosides than include both central and peripheral molecules. It is located on the cell‐facing surface of the N‐terminal domain (NTD) of the Spike protein and it has gradually evolved by mutations to ensure that each SARS‐CoV‐2 variant has a selective kinetics advantage over its predecessors [114]. I propose to name this type of ganglioside‐binding domain type 3 GBD or GBD‐3 (Fig. 3, lower left).

Finally, the study of emerging viruses revealed new aspects of protein‐ganglioside interactions. The recent analysis of the Monkeypox virus allowed to identify a fourth type of ganglioside‐binding domain characterized by a discontinuous annular organization [115]. This ganglioside‐binding domain is conformationally constrained, so that the binding reaction chiefly involves a reorientation of amino acid side chains without modifying the secondary structure of the protein [115]. I propose to name this last ganglioside‐binding domain type 4 GBD, or GBD‐4 (Fig. 3, lower right). Further research is needed to assess whether GBD‐3 and GBD‐4 are present in viruses other than SARS‐CoV‐2 and Monkeypox. In the virology field, GBD‐3 and GBD‐4 are discontinuous epitopes that trigger the production of neutralizing antibodies [92].

Despite clearcut differences in the spatial organization, all four GBDs display common biochemical features, i.e., the presence of key amino acid residues: cationic (Arg or Lys), aromatic (Tyr, Phe, or Trp), and flexible residues (Gly or Ser). Apart from this usual triplet, one or two His residues are often found, especially in GBD‐2, GBD‐3, and GBD‐4 [86]. These combinations ensure an electropositive potential, enough flexibility, H‐bonding, and CH‐π stacking (sugar‐aromatic interactions), all properties essential for ganglioside recognition.

Finally, this proposed classification is obviously schematic, and its main objective is to order in a rational way the different modalities of protein‐ganglioside interactions. In its essence, this classification is above all of structural nature. In this respect, it should be emphasized that our knowledge on the biological functions of gangliosides, especially in the central nervous system, is still incomplete. Phenotypic observations on knock‐out mice lines of GM2/GD2 synthase or GD3 synthase have shed some light on the physiological role of gangliosides [116], but the abnormal phenotypes observed in these knock‐out mouse lines were generally milder than expected, probably because of compensatory mechanisms with remaining glycosphingolipids [117]. The principal abnormalities detected in these studies were inflammatory reactions in the central nervous system [118] and an age‐dependent progressive neurodegeneration [119]. The advantage of the proposed classification is that for each type of GBD, it suggests a molecular mechanism of interaction with a protein, and therefore a possible biological function for the protein‐ganglioside couple.

## Ganglioside‐based therapies: concept and realities

In the preceding section, we have characterized several types of ganglioside‐binding domains including those that mediate the pathological effects of amyloid proteins and virus surface envelope glycoproteins. All these proteins are responsible for infectious or neurodegenerative diseases that require efficient treatments. It is in this context that new approaches targeting gangliosides appear particularly interesting. The proposed classification of ganglioside‐binding domains may simplify the conception of ganglioside‐based molecules intentionally designed as inhibitors of protein‐ganglioside interactions. Given the involvement of gangliosides in human pathologies (Table 1), those drugs are expected to exert beneficial preventive and/or curative effects. On the other hand, gangliosides also fulfill important biological functions, and thus these functions should not be affected by these new drugs. Let us consider the case of GBD‐1.

The binding of a ganglioside to a GBD‐1 domain can be considered as straightforward, as it involves only one ganglioside molecule per protein. However, the binding reaction by itself is quite complex since the ganglioside exerts a chaperone effect on an initially disordered GBD‐1. This chaperone effect is part of a biological mechanism (e.g., membrane receptor regulation [95], receptor endocytosis [120], or exocytosis of synaptic vesicles [77]) that should not be targeted by the therapeutic drug. Thus, whatever the approach selected it should not affect ganglioside‐GBD‐1 interactions. Incidentally, most ganglioside‐binding proteins already interact with gangliosides when they reach the plasma membrane through the secretory pathway [121]. Thus, the ganglioside to which they bind is generally masked by the protein and not accessible to external ligands [122]. Moreover, ganglioside‐cholesterol interactions may influence the accessibility of ganglioside headgroups [122, 123]. Any drug specifically designed to disrupt ganglioside‐GBD‐1 interactions would therefore probably not find its target.

A chimeric synthetic peptide that combines the ganglioside‐binding properties of the GBD‐2 of Alzheimer's β‐amyloid protein (Aβ_1–42_) and Parkinson's disease‐associated α‐synuclein has demonstrated therapeutic efficiency in several *in vitro* and *ex vivo* models of these diseases [34, 124]. This peptide, called AmyP53, has improved ganglioside‐binding properties compared with the proteins from which it is derived [22, 89]. The difficulty to prevent ganglioside‐GBD‐2 interactions is that the structure of both partners evolves during the binding reaction. Aβ_1–42_ and α‐synuclein are intrinsically disordered proteins that tend to adopt mixed turn/α‐helix folding upon binding to raft gangliosides. Once bound to gangliosides, these amyloid proteins penetrate the plasma membrane, then interact with cholesterol which controls the self‐assembly of amyloid proteins [125] and facilitates their oligomerization into Ca^2+^ permeable amyloid pores [109]. The massive entry of Ca^2+^ triggers a neurotoxic cascade involving tau hyperphosphorylation, oxidative stress, and neuronal loss [34, 124]. The involvement of gangliosides (especially GM1), cholesterol, and lipid rafts in the pathogenesis of neurodegenerative diseases, including amyloid pore formation and other mechanisms of membrane damage, has been demonstrated by numerous studies based on a broad range of experimental approaches [125, 126, 127, 128, 129, 130, 131, 132, 133, 134, 135, 136, 137, 138, 139, 140, 141, 142, 143, 144, 145, 146].

The binding of amyloid proteins to lipid raft gangliosides gives us an opportunity to disrupt this neurotoxic cascade at the very first step, and thus to tackle the root cause of Alzheimer's and Parkinson's diseases, i.e., the membrane neurotoxicity of amyloid oligomers [147, 148, 149, 150, 151, 152, 153]. This possibility can be exploited by molecules exhibiting ganglioside‐binding properties [154] able to prevent the access of amyloid proteins to lipid rafts. This is the case of AmyP53 that has been shown to adapt its structure to the flexible and moving conformations of raft gangliosides [22]. AmyP53 blocks the binding of amyloid proteins to lipid rafts through an unprecedent mechanism of interaction with gangliosides, defining a new class of therapeutic agents coined adaptative peptides. AmyP53 crosses the blood–brain barrier after intravenous injection, and it can also reach the brain after intranasal administration [155]. The design of AmyP53 opens the route for new therapeutic strategies for the patients with Alzheimer's or Parkinson's disease and for a pipeline of antiganglioside drugs based on the same basic principles.

From a stochiometric point of view, GBD‐3 and GBD‐4 recognize more raft gangliosides than GBD‐1 and GBD‐2. This situation must be considered for the design of new antiviral drugs. It can be assumed that to occupy the raft surface recognized by these large GBDs, it is necessary to reach a high concentration of antiviral compounds at the level of the infection sites. A simple way to increase the local concentration of the drugs is to use multimers, which can be based either on synthetic peptides [156, 157, 158] or sugar derivatives [159, 160, 161].

Alternatively, why not directly using gangliosides as therapeutic drugs [21]? Indeed, GM1 has been demonstrated to exert a neuroprotective effect, which is justified to test it for the treatment of neurological disorders including Parkinson's disease [29, 45, 162, 163]. One identified drawback is the fact that gangliosides do not efficiently penetrate the blood–brain barrier (BBB) [164]. An interesting alternative is to use the soluble and hydrophilic GM1‐oligosaccharide (OligoGM1), which is able to cross the human BBB more efficiently than GM1 [165]. In this regard, one might wonder whether the fact of using either the ganglioside GM1 [162] or an antiganglioside peptide (AmyP53) [22] to treat neurodegenerative diseases is not contradictory. In fact, any mechanism able to prevent the binding of amyloid proteins to membrane gangliosides would prevent amyloid pore formation, membrane damage, and downstream Ca^2+^ neurotoxicity. Whatever the therapeutic mechanism of exogenous GM1, one could consider that exogenous GM1 micelles could bind to amyloid proteins and neutralize their oligomerization, maintaining them outside the plasma membrane. AmyP53 can block amyloid pore formation by preventing amyloid monomers and oligomers to interact with the plasma membrane. Thus, collectively, all these innovative approaches enrich our arsenal of ganglioside‐based therapeutic agents. Finally, due to its prominent role in lipid raft homeostasis, cholesterol is considered as a promising therapeutic opportunity for the treatment of cancer [166] and neurodegenerative diseases [167]. Such therapies may exert indirect effects on ganglioside organization in lipid rafts [168], which suggests the use of cholesterol‐affecting drugs such as cyclodextrins as an indirect ganglioside‐targeting therapy.

## Sialic acid biochemistry and translational neurosciences

It is particularly frustrating that numerous promising drugs for neurodegenerative diseases, that were based on work in rodent models, failed in clinical trials [169]. This raises the question of the real need of animal experiments for human brain diseases [170]. These failures may be due to various parameters including irrelevant animal models selected on the basis of a wrong target or different biodistribution of the medicine in rodents and humans. However, in addition to these potential caveats, there is a characteristic of human brain gangliosides that needs careful consideration, especially for pathological mechanisms controlled by gangliosides. Two types of sialic acids exist in mammals, with a distinct biochemical structure: *N*‐acetylneuraminic acid (Neu5Ac) and *N*‐glycolylneuraminic acid (Neu5Gc) [86]. Humans express only Neu5Ac, whereas other mammalian species express both Neu5Ac and Neu5Gc [171]. A side consequence is that high‐meat diets induce the production of anti‐Neu5Gc antibodies, which may be associated with an increased risk for cancer [172]. Interestingly, rabbit is the animal that expresses the lowest amounts of Neu5Gc [172]. What is particularly critical for assessing the efficiency and safety of a ganglioside‐directed treatment is the sialic acid content of gangliosides in the brain of the selected animal models. Indeed, a protein that binds to a ganglioside containing Neu5Ac (e.g., human GM1) will not necessarily recognize this ganglioside if its sialic acid is Neu5Gc. Biochemical analysis revealed that rabbits and rats express almost exclusively Neu5Ac in their brain gangliosides [173, 174]. Hence, they are relevant models for studying the effect of drugs that target human brain gangliosides. On the other hand, dogs [175], pigs [172, 175], and most monkeys (including Old World species that are widely used as animal models such as vervets, rhesus, and cynomolgus [176]) express significant amounts of Neu5Gc in the brain [177]. These animal species are thus not relevant for testing antiganglioside therapies. Now, if we consider peripheral tissues, rabbit is again the best alternative for nonrodent species [172]. In summary, rats and rabbits are the species that should be preferred for regulatory toxicology and proof‐of‐concept studies of drugs targeting gangliosides. The development of new animal models (or any alternative) taking into account the expression of Neu5Ac together with the absence of Neu5Gc will most likely increase the reliability of translational neurosciences. Most importantly, these models should be based on amyloid oligomers, which are now considered as the most neurotoxic species in Alzheimer's and Parkinson's disease [147, 178].

## Conclusion

We are at the very beginning of the development of therapeutic molecules specifically designed to target gangliosides. These new drugs will make it possible to propose new strategies to prevent and treat many diseases in which gangliosides are involved, including cancer, diabetes, bacterial, virus, parasite, and prion infections, and neurodegenerative diseases. In many cases, we have been able to demonstrate the interaction of a protein involved in these diseases (‘the culprit’) with one or more gangliosides (‘the accomplices’) located in a lipid raft. Thus, these diseases should be considered as membrane disorders. The proteins that bind to these gangliosides have at their disposal a repertoire of ganglioside‐binding domains, which includes, to date, four types of domains which I propose to name and classify from GBD‐1 to GBD‐4. It is likely that this classification will be gradually enriched with new entries. We will have to take into consideration the biochemical characteristics of each of these domains in order to develop a therapeutic arsenal adapted to the expression of each targeted ganglioside according to the pathology with which it is associated.

## Conflict of interest

JF is the co‐inventor of the AmyP53 peptide (patent Application EP15709163.8A), currently under development for the treatment of Alzheimer's and Parkinson's diseases by the AmyPore company.

## Author contributions

JF is the unique author of this article. JF wrote the article and prepared the figures. JF perfomed the bibliography analysis and wrote the article on the basis of his own expertise on gangliosides and lipid rafts in the context of human diseases.
